# Combining Bioinformatics Techniques to Study the Key Immune-Related Genes in Abdominal Aortic Aneurysm

**DOI:** 10.3389/fgene.2020.579215

**Published:** 2020-12-10

**Authors:** Han Nie, Jiacong Qiu, Si Wen, Weimin Zhou

**Affiliations:** ^1^Department of Vascular Surgery, The Second Affiliated Hospital of Nanchang University, Nanchang, China; ^2^Divison of Vascular Surgery, The First Affiliated Hospital, Sun Yat-sen University, Guangzhou, China; ^3^Xinjian District People’s Hospital of Jiangxi Province, Jiangxi, China

**Keywords:** immune related genes, abdominal aortic aneurysm, bioinformatics, vascular, surgery

## Abstract

Approximately 13,000 people die of an abdominal aortic aneurysm (AAA) every year. This study aimed to identify the immune response-related genes that play important roles in AAA using bioinformatics approaches. We downloaded the GSE57691 and GSE98278 datasets related to AAA from the Gene Expression Omnibus database, which included 80 AAA and 10 normal vascular samples. CIBERSORT was used to analyze the samples and detect the infiltration of 22 types of immune cells and their differences and correlations. The principal component analysis showed significant differences in the infiltration of immune cells between normal vascular and AAA samples. High proportions of CD4^+^ T cells, activated mast cells, resting natural killer cells, and 12 other types of immune cells were found in normal vascular tissues, whereas high proportions of macrophages, CD8^+^ T cells, resting mast cells, and six other types of immune cells were found in AAA tissues. In the selected samples, we identified 39 upregulated (involved in growth factor activity, hormone receptor binding, and cytokine receptor activity) and 133 downregulated genes (involved in T cell activation, cell chemotaxis, and regulation of immune response mediators). The key differentially expressed immune response-related genes were screened using the STRING database and Cytoscape software. Two downregulated genes, *PI3* and *MAP2K1*, and three upregulated genes, *SSTR1, GPER1*, and *CCR10*, were identified by constructing a protein–protein interaction network. Functional enrichment of the differentially expressed genes was analyzed, and the expression of the five key genes in AAA samples was verified using quantitative polymerase chain reaction, which revealed that *MAP2K1* was downregulated in AAA, whereas *SSTR1, GEPR1*, and *CCR10* were upregulated; there was no significant difference in PI3 expression. Our study shows that normal vascular and AAA samples can be distinguished via the infiltration of immune cells. Five genes, *PI3, MAP2K1, SSTR1, GPER1*, and *CCR10*, may play important roles in the development, diagnosis, and treatment of AAA.

## Introduction

With changes in lifestyle, the incidence rates of cardiovascular and cerebrovascular diseases have increased every year, making them a serious public health problem. According to World Health Organization statistics, cardiovascular disease is the leading cause of death worldwide. In 2011, coronary heart disease and cerebrovascular disease caused 13.2 million deaths, accounting for 24% of the total global deaths. It is estimated that by 2030, the number of deaths due to cardiovascular diseases will increase to 23.3 million, and cardiovascular diseases will continue to be the leading cause of death (World Health Organization, 2013). Reportedly, approximately 13,000 people die of abdominal aortic aneurysm (AAA) every year (Rooke et al., 2012).

Current studies suggest that age (more than 65 years), family history, sex (male), and smoking are important risk factors for AAA (Sakalihasan et al., 2018). AAA has become an important cause of death in the elderly who are more than 65 years old and is a serious aortic disease involving irreversible radial dilatation of the abdominal aorta more than 3 cm or 1.5 times the normal diameter due to various reasons (Umebayashi et al., 2018). Most AAA patients are asymptomatic and cannot be treated before the tumor ruptures, or the patient dies (Golledge and Norman, 2011). Aneurysm rupture is an important cause of mortality in patients with AAA (Golledge et al., 2006), which is reportedly as high as 80%. Open surgery and interventional surgery are the main methods to treat AAA. However, they have limited use because the prevalence of AAA is positively correlated with age, and patients with AAA often suffer from other cardio-cerebrovascular diseases, such as heart failure, atherosclerosis, and ischemic cardiomyopathy (Arya et al., 2015).

Studies have shown that if the increase in the aortic diameter can be slowed down by 50%, the annual rates of aortic reconstruction surgery for AAA can be halved. In clinical practice, only patients whose AAAs are greater than 5.5 cm and who are at risk of rupture are treated with open surgery (Golledge et al., 2006), and there is no drug to slow down the development of AAA. The occurrence and development of AAA is a complex process involving multiple factors. It is generally believed that AAA is directly related to atherosclerosis, hypertension, chronic obstructive pulmonary disease, and a variety of proteases, but there is no clear evidence to explain the roles of these factors in the pathogenesis of AAA. The pathophysiological processes of AAA include infiltration of inflammatory cells (Moxon et al., 2010; Gordon and Toursarkissian, 2014), degradation of elastic and collagen fibers, death of smooth muscle cells, defects of the arterial wall, and increased oxidative stress (Newman et al., 1994).

Vascular inflammation is the first event in the development of AAA. In the early stages of the disease, immune cells such as lymphocytes, macrophages, mast cells, neutrophils, and natural killer (NK) cells infiltrate and accumulate in the blood vessels and surrounding tissues, causing a series of inflammatory reactions in the vascular wall (Eliason et al., 2005; Forester et al., 2006; Rateri et al., 2011; Wang et al., 2014a; Yan et al., 2016). The invasion of inflammatory cells often stimulates smooth muscle cells to secrete matrix metalloproteinases, which degrade elastin and collagen, thus reducing the stability of the arterial wall and inducing the apoptosis of vascular smooth muscle cells (Rizzo et al., 1989; Sakalihasan et al., 1996; Culav et al., 1999; Longo et al., 2002, 2005; Keeling et al., 2005). Inflammatory response plays an important role in the immune system and is involved in the occurrence and development of AAA (Yamaguchi et al., 2000; Liu et al., 2015). Indeed, lowering interleukin (IL)-17 levels in animal models slows down the increase in aortic diameter in aneurysms (Chang et al., 2015). Therefore, this study aimed to analyze the infiltration of immune cells in AAA and identify genes related to the immune response that play a role in the development of AAA.

## Materials and Methods

### Data Acquisition and Processing

We searched the Gene Expression Omnibus database^1^ and obtained two gene expression datasets, GSE57691 (including 49 AAA samples and 10 normal aortic vessels samples) (Biros et al., 2015) and GSE98278 (Gäbel et al., 2017) (including 31 AAA samples), which included 80 samples from AAAs and 10 samples from normal aortic vessels. We used Limma (Ritchie et al., 2015) and SVA (Leek et al., 2019) packages in R (3.61) to correct the sample data.

### Infiltration of Immune Cells in the Samples

To investigate the infiltration of immune cells in AAAs and normal aortic vessels and evaluate and predict the enrichment of immune cells in the samples, we used CIBERSORT (Newman et al., 2015). CIBERSORT is a tool used to deconvolute the expression matrix of immune cell subtypes based on the principle of linear support vector regression. RNA-Seq data were used to estimate the infiltration of immune cells. CIBERSORT analyzed the relative abundance of 22 types of immune infiltrating cells in each sample, including NK cells, T cells, B cells, and macrophages. The 69 samples were screened according to the *P*-value predicted by CIBERSORT (*P* < 0.05).

### Principal Component Analysis

We reduced the dimensions of the samples and performed principal component analysis (PCA) of the infiltration of 22 types of immune cells in the samples.

### Acquisition of Immune Response-Related Gene Expression Profiles

From ImmPort^2^, we downloaded 2,498 immune response-related genes, including those related to antigen-presenting cells, chemokines and their receptors, cytokines and their receptors, interferons, and ILs. We used the Limma package (Ritchie et al., 2015) in R (3.61) to compare gene expression data of immune response-related genes in AAA and normal aortic blood samples downloaded from the Gene Expression Omnibus database and extracted information on the expression levels of immune response-related genes in the samples.

### Screening of Differentially Expressed Immune Response-Related Genes

After the data were standardized using the Limma software package (Ritchie et al., 2015) in R, we identified immune response-related, differentially expressed genes by comparing normal aortic blood vessel samples with AAA samples. If the change factor was greater than onefold (|fold change| ≥ 1) and the corrected *P*-value (false discovery rate) ≤ 0.05, the gene was considered to be differentially expressed.

### Construction and Analysis of Protein–Protein Interaction Network

We used the STRING^3^ database to construct a protein–protein interaction (PPI) network of the differentially expressed gene products. The PPI file was imported to Cytoscape 3.6.0^4^, and the MCODE plug-in was used to map the PPIs. The degree, closeness, intermediate degree of each node in the network, and the average value of each protein’s nodal degree were defined as the threshold of the PPI network nodes, and the proteins whose degrees were greater than the threshold value were selected. The key nodes of the PPI network were identified, and the correlation scores of the nodes and their interacting proteins were calculated.

### Gene Ontology and Kyoto Encyclopedia of Genes and Genomes Enrichment Analyses

Gene ontology and Kyoto Encyclopedia of Genes and Genomes enrichment analyses were performed using clusterProfiler (Yu et al., 2012) package in R (3.6.1). The hypergeometric distribution was used to analyze and calculate the significance levels of these differentially expressed genes in each signaling pathway to identify the signaling pathways that were significantly affected (*P* < 0.05).

### Identification of Differentially Expressed Immune Response-Related Genes

From June 2019 to February 2020, we recruited eight patients with AAA who underwent surgical resection in the Second Affiliated Hospital of Nanchang University and Sun Yat-sen University. All tissue samples were frozen in liquid nitrogen during the surgery. Two experienced pathologists confirmed AAA. This study was approved by the Ethics Committee of the Second Affiliated Hospital of Nanchang University and Sun Yat-sen University. Written informed consent was obtained from each patient before surgery. The samples were pretreated, and RNA was extracted with TRIzol reagent (German DBI) for real-time quantitative polymerase chain reaction (qPCR). Reverse transcription of RNA into complementary DNA was performed using the Bestar qPCR RT Kit (DBI, Germany) following the manufacturer’s instructions. qPCR was performed to determine the expression levels of *PI3, MAP2K1, SSTR1, GPER1, CCR10, IL-6, IL-17*, tumor necrosis factor *(TNF)-*α, and *ACTB* (internal reference gene) in the samples. Primer sequences were obtained from PrimerBank^5^ (Table 1).

**TABLE 1:** Gene sequence.

	Forward primer	Reverse primer
PI3 (5′ -> 3′)	CACGGGAGTTCCTGTT AAAGG	TCTTTCAAGCAGCGGT TAGGG
MAP2K1 (5′ -> 3′)	TGTCGCCAGAAAGAC TCCAG	TCCATTCCGTATGAGC TAAGGG
SSTR1 (5′ -> 3′)	CCAGCATCTACTGTCTG ACTGT	ATGACGAGCAGCGA TAGCAC
GPER1 (5′ -> 3′)	CCTGCTTCTGTTTCG CGGAT	CAATGAGGGAGTAGC ACAGGC
CCR10 (5′ -> 3′)	GCAAACGCAAGGAT GTCGC	CGTAGAGAACGGGATT GAGGC
β-actin (5′ -> 3′)	ATCGTGCGTGACATTAA GGAGAAG	AGGAAGGAAGGCTGGAA GAGTG

### Statistical Analysis

Data were analyzed using GraphPad Prism 7 (GraphPad Software Inc.) and IBM SPSS 17.00 (IBM Analytics, United States). Chi-square and Fisher’s exact tests were used for the qualitative analysis of the data. Data were expressed as the mean ± standard deviation (x ± s) and compared using Student’s *t*-test. PCR data were analyzed using GraphPad Prism 8. *P* < 0.05 was considered as statistically significant and *P* < 0.01 as very significant.

## Results

### Infiltration of Immune Cells

**FIGURE 1:**
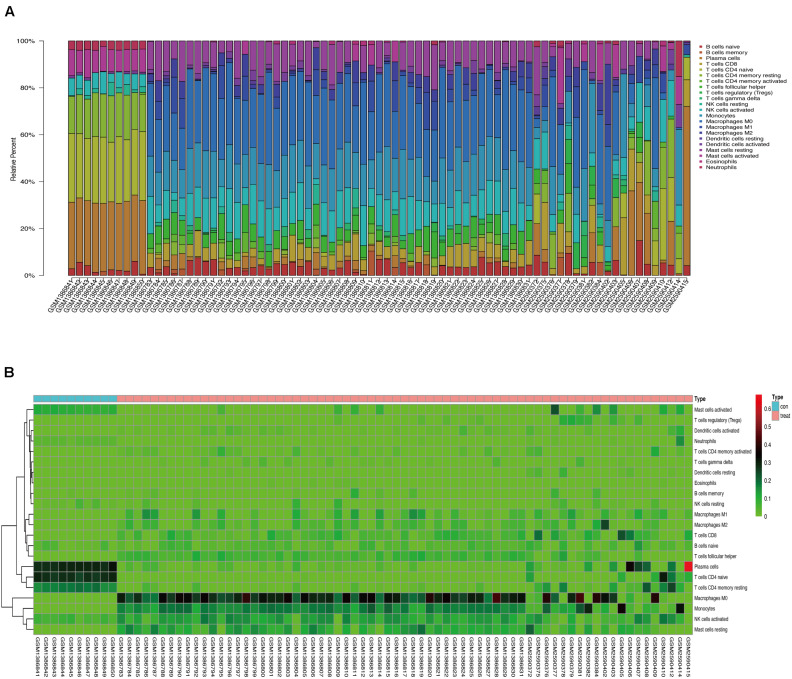
**(A)** Proportion of 22 kinds of immune cells in the chip. **(B)** Expression heat map of differential immune cells in the sample.

**FIGURE 2:**
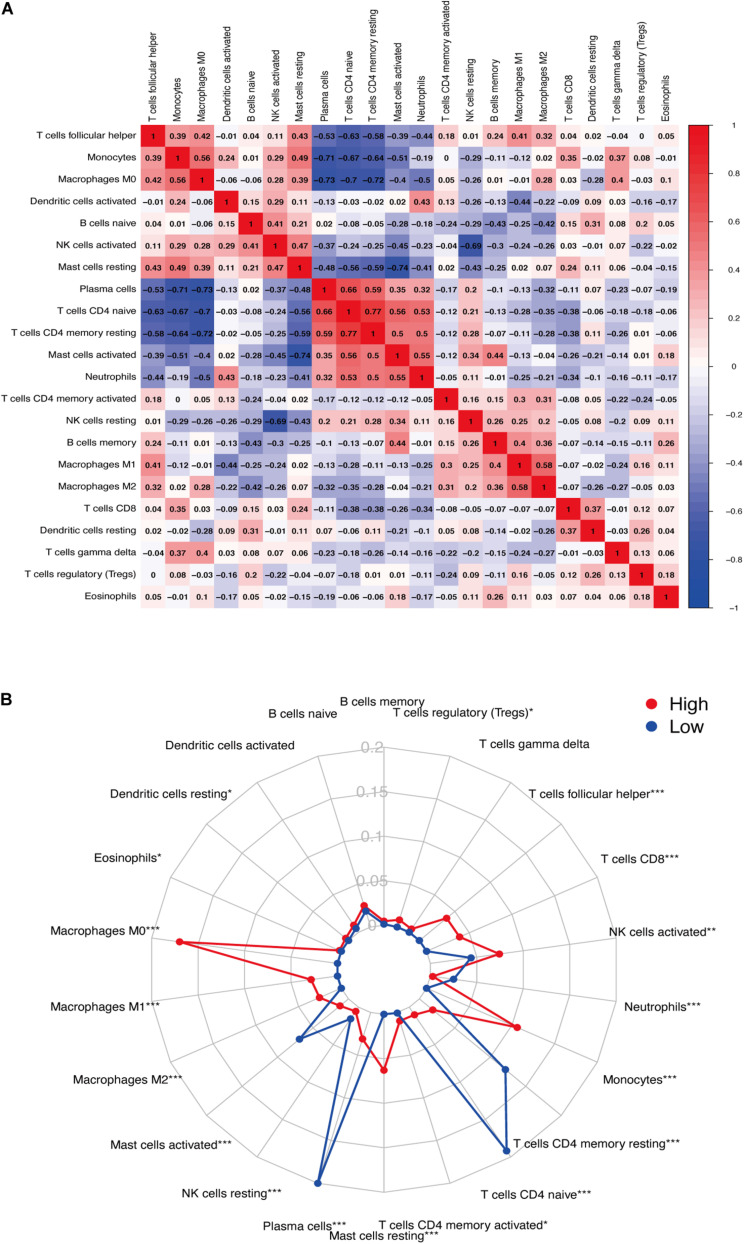
**(A)** Correlation between immune cells. **(B)** Infiltration of immune cells (*P < 0.05; **P < 0.01; ***P < 0.001).

**FIGURE 3:**
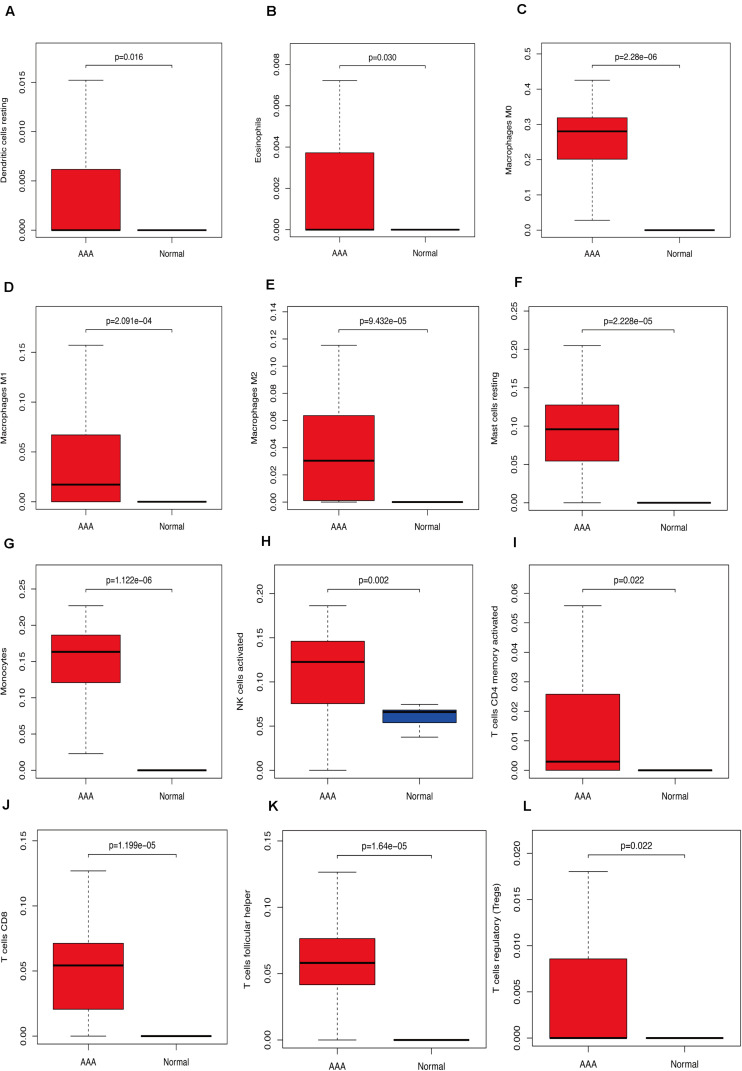
Immunocyte expression ratio, red represents the expression of immune cells in abdominal aortic aneurysm, and blue represents the expression of immune cells in normal vascular tissues. **(A)** The expression of resting dendritic cells. **(B)** The expression of eosinophils. **(C)** The expression of macrophages M0. **(D)** The expression of macrophages M1. **(E)** The expression of macrophages M2. **(F)** The expression of resting mast cells. **(G)** The expression of monocytes, **(H)** Activated NK cell expression, **(I)** activated CD4 memory cell expression, **(J)** CD8 cell expression, **(K)** follicular helper cell expression, **(L)** T regulatory cell (Tregs) expression.

**FIGURE 4:**
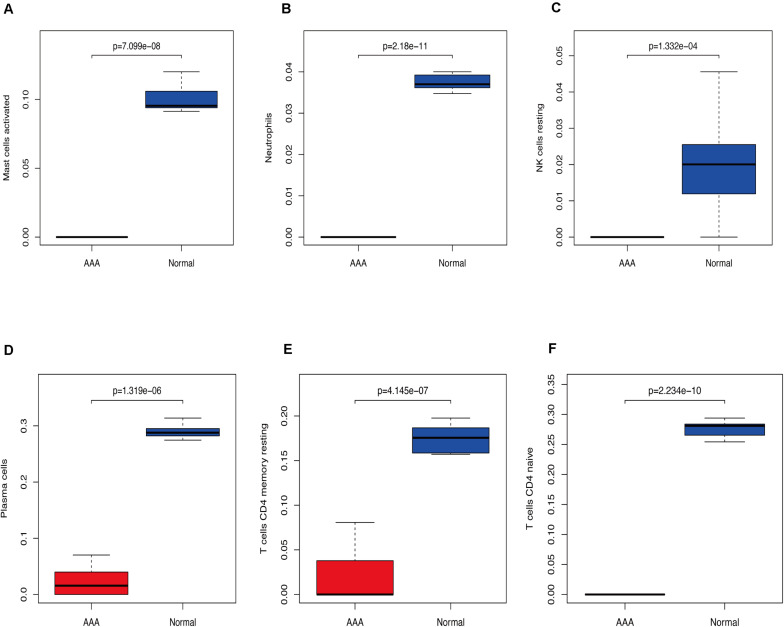
Immunocyte expression ratio, red represents the expression of immune cells in abdominal aortic aneurysm, and blue represents the expression of immune cells in normal vascular tissues **(A)** Expression of activated mast cells. **(B)** Expression of neutrophils. **(C)** The expression of resting NK cells. **(D)** Expression of plasma cells. **(E)** Expression of resting CD4 memory cells. **(F)** Expression of CD4 naïve cells.

We used CIBERSORT to analyze immune cell infiltration in the samples and selected 69 AAA samples and 10 normal aortic samples that met the standard according to the analysis (*P* < 0.05). We found a significant difference in the abundance of 22 types of immune cells in the 10 normal aortic samples and 69 AAA samples (Figure 1A). There were also significant differences in the infiltration ratio of plasma cells, T cells, naïve CD4^+^ T cells, resting memory CD4^+^ T cells, and macrophages M0 in normal aortic and AAA samples (Figure 1B). Correlation analysis of the 22 types of immune cells revealed that the negative correlation between resting mast cells and activated mast cells was the strongest and that between macrophages M0 and plasma cells, macrophages M0 and resting memory CD4^+^ T cells, and monocytes and plasma cells was strong. A negative correlation was observed between resting CD4^+^ memory T cells and naïve CD4^+^ T cells and between plasma cells and CD4^+^ T cells; a strong positive correlation was observed between naïve CD4^+^ T cells (Figure 2A). Figure 2B clearly shows that there are 18 types of immune cells with different proportions in normal and AAA samples, such as resting dendritic cells (Figure 3A), eosinophils (Figure 3B), macrophages M0 (Figure 3C), macrophages M1 (Figure 3D), macrophages M2 (Figure 3E), resting mast cells (Figure 3F), monocytes (Figure 3G), activated NK cells (Figure 3H), activated CD4^+^ memory T cells (Figure 3I), CD8^+^ T cells (Figure 3J), and volatile T helper cells (Figure 3K). The proportion of regulatory T cells (Figure 3L) was increased in AAA samples, whereas the proportions of activated mast cells (Figure 4A), neutrophils (Figure 4B), resting NK cells (Figure 4C), plasma cells (Figure 4D), resting memory CD4^+^ T cells (Figure 4E), and naïve CD4^+^ T cells (Figure 4F) were increased in normal aortic samples.

### Principal Component Analysis of Samples

**FIGURE 5:**
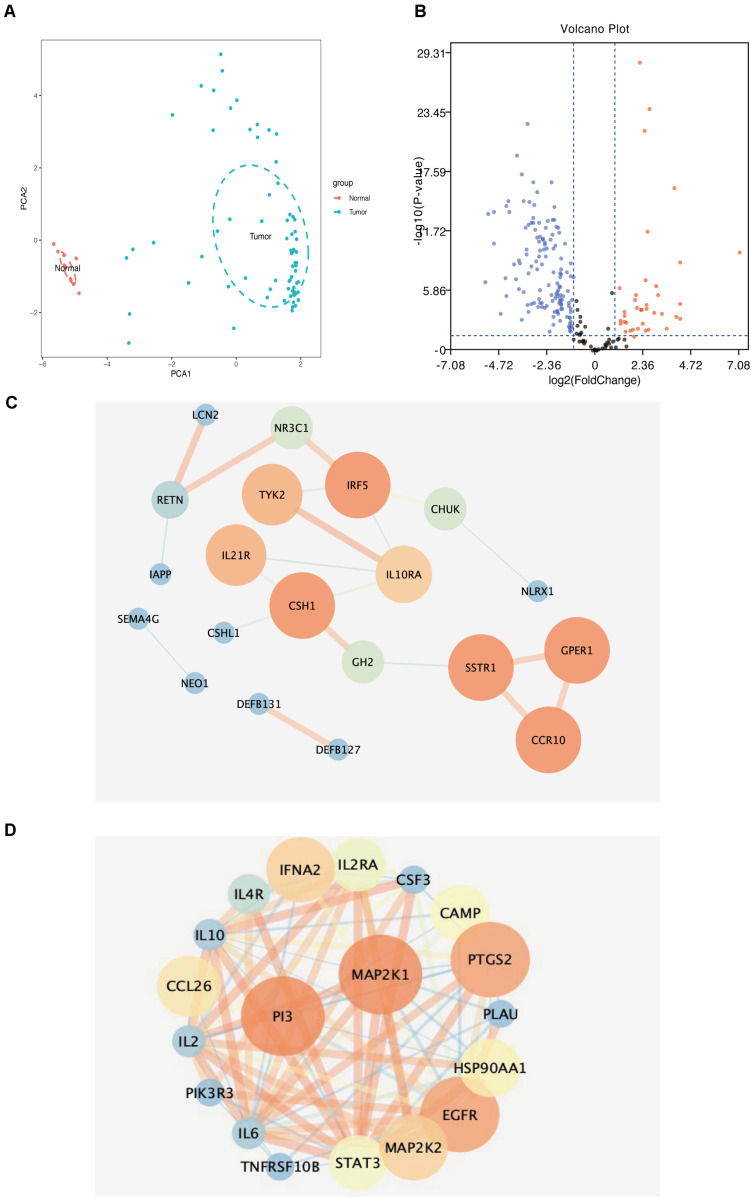
**(A)** Principal component analysis distribution of normal aortic samples and abdominal aortic aneurysm samples. **(B)** Volcanic map of differential immune-related genes in samples (blue dots represent downregulated genes, red represents core genes in immune-related genes downregulated by upregulated genes). **(C)** Downregulated immune-related genes (the darker the color, the larger the circle, the higher the score of immune-related genes). **(D)** Upregulated immune-related genes (the darker the color, the larger the circle, the higher the score of immune-related genes).

We reduced the dimensions of the samples and performed PCA based on the infiltration of 22 types of immune cells in the samples. The results showed that normal aortic and AAA samples could be clearly distinguished based on the infiltration of immune cells (Figure 5A).

### Differentially Expressed Immune-Related Gene Screening

We extracted and analyzed differences in the expression of immune response-related genes in 69 AAA and 10 normal aortic samples. The results showed 39 upregulated and 133 downregulated immune response-related genes (Figure 5B).

### Construction and Analysis of the Protein–Protein Interaction Network

We uploaded 39 upregulated and 133 downregulated genes into the STRING database (Supplementary Figures 1A,B) to construct the PPI network. Then, we imported the PPI network into Cytoscape and used MCODE to identify the related nodes. As shown in Figures 5C,D, the larger the degree of the node in the graph, the darker the color and the larger the diameter of the node. Among the upregulated genes, *SSTR1, GPER1*, and *CCR10* had the largest nodes (highest degrees), whereas, among the downregulated genes, *PI3* and *MAP2K1* had the largest nodes (highest degrees) (Table 2).

**TABLE 2:** Key of the immune related genes.

Symbol	Category	
SSTR1	Cytokine_Receptors	
GPER1	Cytokine_Receptors	
CCR10	Antimicrobials	
PI3	Antimicrobials	
MAP2K1	NaturalKiller_Cell_Cytotoxicity	

**Name**	**Score**	**Name**	**Score**

CSH1	6	MAP2K1	8.59090909
GPER1	6	PI3	8.59090909
CCR10	6	PTGS2	8.41666667
IRF5	2	EGFR	8.41666667
SSTR1	2	IFNA2	8
TYK2	1.66666667	MAP2K2	8
IL21R	1.66666667	CCL26	7.82222222
IL10RA	1.4	HSP90AA1	7.71428571
NR3C1	0.66666667	CAMP	7.64444444
GH2	0.66666667	STAT3	7.60233918
CHUK	0.66666667	IL2RA	7.56363636
RETN	0.5	IL4R	7.27272727
LCN2	0	IL10	7.0952381
DEFB127	0	IL2	7.0952381
DEFB131	0	IL6	7.0952381
CSHL1	0	CSF3	7.01470588
IAPP	0	PLAU	7
NEO1	0	TNFRSF10B	7
SEMA4G	0	PIK3R3	7
NLRX1	0		

### Gene Ontology and Kyoto Encyclopedia of Genes and Genomes Enrichment Analyses

**FIGURE 6:**
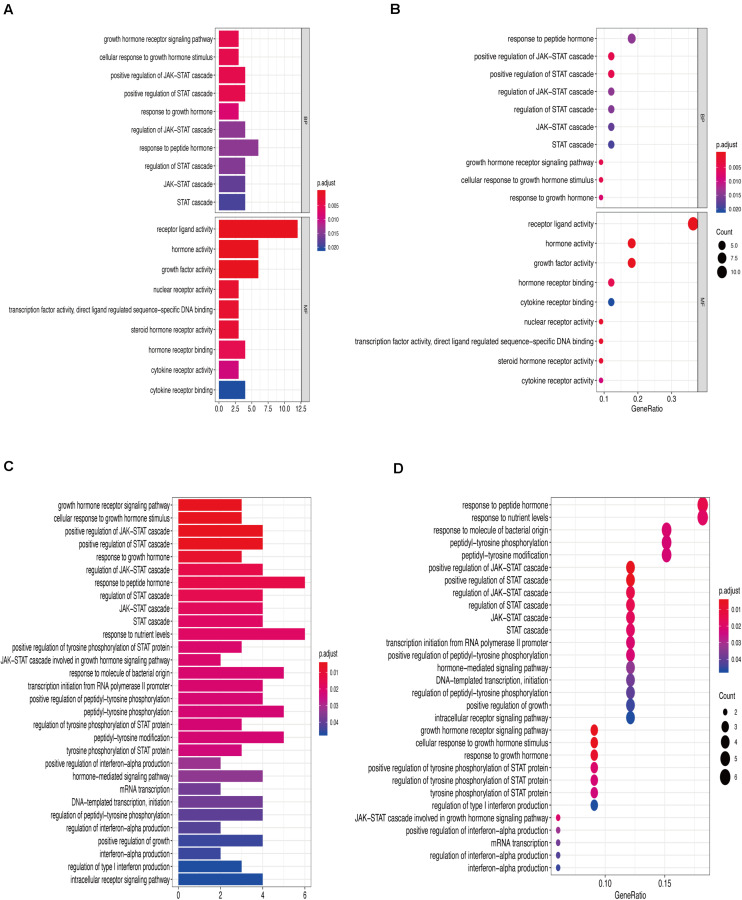
**(A)** Gene Ontology enrichment analysis histogram of upregulated immune-related genes. **(B)** Upregulation of immune-related genes, Gene Ontology enrichment analysis histogram. **(C)** Upregulation of immune-related genes, Kyoto Encyclopedia of Genes and Genomes enrichment analysis histogram. **(D)** Upregulation of immune-related genes, Kyoto Encyclopedia of Genes and Genomes enrichment analysis histogram.

**FIGURE 7:**
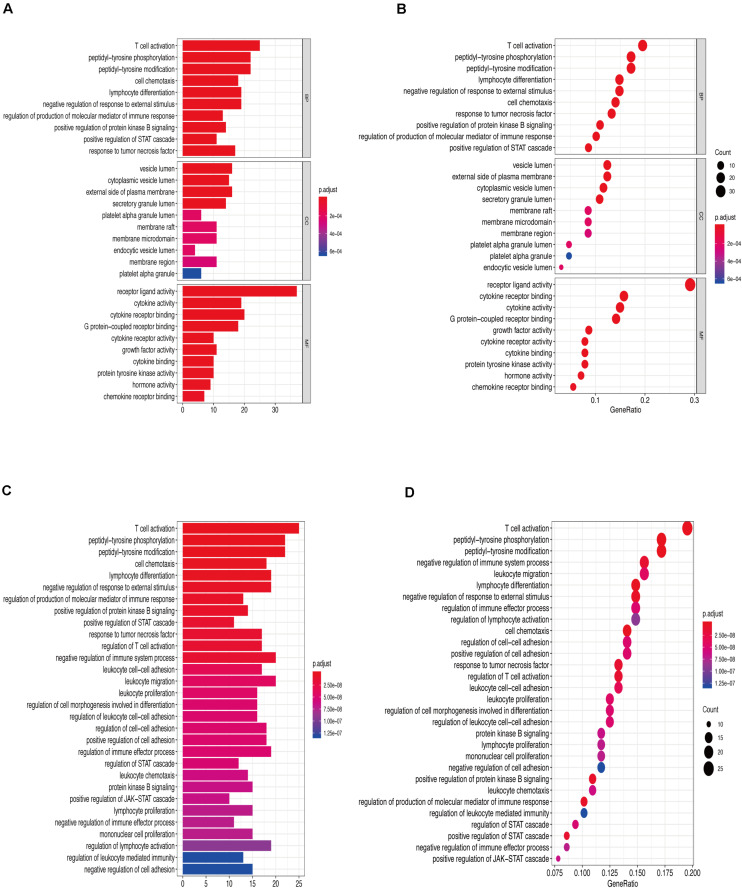
**(A)** Downregulation of immune-related genes, Gene Ontology enrichment analysis histogram. **(B)** Downregulation of immune-related genes, Gene Ontology enrichment analysis histogram. **(C)** Downregulated immune-related genes Kyoto Encyclopedia of Genes and Genomes enrichment analysis histogram. **(D)** Downregulated immune-related genes Kyoto Encyclopedia of Genes and Genomes enrichment analysis dot map.

Figures 6A,B show that the biological processes regulated by the upregulated immune-response-related genes include response to growth hormone, peptide hormone response, growth factor activity, and cytokine receptor activity. These processes were closely related to the growth hormone response, positive regulation of the JAK-STAT cascade, growth hormone receptor signaling, regulation of tyrosine phosphorylation of STAT proteins (Figures 6C,D), and hormone-mediated signaling. Figures 7A,B show that the downregulated genes were mainly involved in T cell activation, peptide tyrosine phosphorylation, peptidyl tyrosine modification, negative regulation of external stimulus-response, response to TNF, cell chemotaxis, as well as regulation of immune response molecular mediators, platelet α granules, and protein tyrosine kinase activity. This was related to T cell activation, TNF-mediated positive regulation of STAT signaling cascade, negative regulation of external stimulation response, positive regulation of protein kinase B signal transduction, leukocyte migration, leukocyte proliferation, regulation of cell morphogenesis during differentiation, regulation of STAT cascade, leukocyte chemotaxis, and lymphocyte proliferation (Figures 7C,D).

### Identification of Differentially Expressed Immune Genes

**FIGURE 8:**
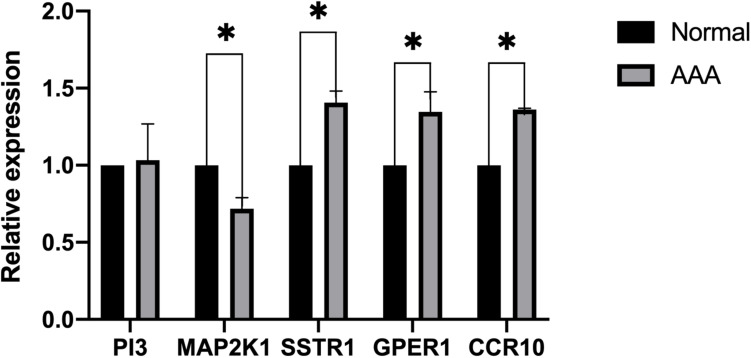
Expression of five immune-related key genes in the samples was analyzed using PCR (^∗^*P* < 0.05).

We collected eight AAA samples and six samples from the adjacent aortic aneurysm vessels at the Second Affiliated Hospital of Nanchang University. The expression levels of SSTR1, GPER1, CCR10, PI3, and MAP2K1 were measured using qPCR. The relative expression of each target gene in Figure 5 was calculated as follows: relative expression = 2 – Δ CT, where Δ CT = CT value of target gene – CT value of the internal reference gene (actin). Compared with normal samples, the expression levels of SSTR1, GPER1, and CCR10 were increased, whereas those of MAP2K1 were decreased in AAA samples; no significant difference was observed in the expression of PI3 (Figure 8). We found that the expression of vascular inflammatory factors IL-6, IL-17, and TNF-α increased significantly in AAA samples (Supplementary Figure 2).

## Discussion

Abdominal aortic aneurysm has always been the focus of vascular surgery research. Due to second-generation sequencing development, more and more researchers began to use bioinformatics technology to study AAA. Wang et al. thought that UBB, NFIA, sparcl1, and other genes play an important role in AAA by comparing gene expression levels and simply constructing a regulatory network (Wang et al., 2018). Gan et al. found that hsa-mir-30a-gng2 and hsa-mir-15b-acss2 may play a role in the development of AAA by screening differentially expressed miRNAs and mRNA and predicting the regulatory relationship through a database (Gan et al., 2019). Gäbel et al. (2017) showed that ccl4l1 and ANGPTL4 were closely related to AAA rupture. In addition, according to Biros et al. (2015), small AAA and large AAA have some unique immune characteristics. They found that cytotoxic T lymphocyte-associated protein 4 (CTLA4) was upregulated in small AAA, and CD8a was upregulated in large AAA. Subsequent studies found that the downregulation of CTLA4 could promote the immune response induced by T cells, leading to the occurrence of AAA. However, few studies have used bioinformatics technology to comprehensively analyze the infiltration of immune cells in AAA. Only one article analyzes the infiltration of immune cells in a ruptured abdominal aortic aneurysm and stable abdominal aortic aneurysm. The sample size is only 48 cases, and there is no laboratory data validation (Lei et al., 2020).

In this study, we analyzed the GSE57691 and GSE98278 datasets related to AAAs. A total of 90 samples were included to comprehensively analyze the status of immune cell infiltration in AAA and the correlation between immune cells. Furthermore, we screened the genes that play a key role in AAA by constructing a network and verified the results by collecting clinical samples for PCR analysis, which made our research results more reliable. We found a significant difference in the infiltration ratio of 22 types of immune cells between normal aortic samples and AAA samples (Figure 1A). Furthermore, we found that the proportions of cells related to M1 macrophages, M2 macrophages, mast cell quiescence, monocytes, NK cell activation, and activated memory CD4^+^ T cells increased in AAAs, whereas those related to mast cell activation, NK cell quiescence (Figure 4C), quiescent memory CD4^+^ T cells (Figure 4E), and naïve CD4^+^ T cells (Figure 4F) increased in normal aortic samples. Although the role of mast cells in AAAs is not clear (Sillesen et al., 2015), the recruitment of macrophages in aortic tissue marks the beginning of infiltration into the adventitia, which promotes the secretion of matrix degradants to contribute to the formation of AAAs (Blomkalns et al., 2013). Activated memory CD4^+^ T cells can play a pro-inflammatory role by differentiating into Th2 cells, and studies have shown that the proportion of Th2 cells in AAAs is increased (Wang et al., 2014b). Furthermore, NK cells can produce pro-inflammatory factors, such as IL-2 and interferon-γ, which can lead to increased cytotoxic activity and promote AAA formation (Chan et al., 2005a, b; Forester et al., 2006). In addition, there is evidence that monocyte depletion can inhibit the formation of AAAs (Wang et al., 2010). Moreover, the analysis of these same datasets also showed that the invasion of monocytes and CD4T cells into the vascular wall and expression of cytotoxic mediators might be the cause of AAA (Ritchie et al., 2015; Gäbel et al., 2017). Thus, our results are highly consistent with these findings. The results of the PCA analysis showed that normal aortic and AAA samples could be clearly distinguished by the infiltration of immune cells. Also, our study confirmed that the expression of inflammatory factors IL-6, IL-17, and TNF was significantly increased in abdominal aortic aneurysm samples compared with adjacent aorta samples. Therefore, searching for the key immune-related genes in AAA is a new direction to study the occurrence and development of AAA.

We screened 172 immune-response-related genes that were differentially expressed in normal aortic vascular samples and AAA samples, including 39 upregulated genes and 133 downregulated genes. These genes were used to construct a PPI network. *SSTR1*, *GPER1*, and *CCR10* were found to be important genes in the upregulated gene network, whereas *PI3* and *MAP2K1* were important genes in the downregulated gene network. SSTR1, one of the five somatostatin receptors (SSTRs), plays an important role in neuroendocrine tumors, such as lung carcinoid (Vesterinen et al., 2019), and the overexpression of SSTR1 can inhibit cell proliferation (Zou et al., 2015). Compared with normal uterine tissues, the expression of GPER1 in uterine leiomyoma is higher and increases cell migration (Kim et al., 2020), which may be one reason for its high expression in AAAs. CCR10 is an important receptor-mediating chemokine, which participates in angiogenesis by endothelial cells. It can promote angiogenesis and improve wound healing by inhibiting the reaction between CCL28 and CCR10 (Chen et al., 2020). CCR10 is expressed in the skin of most patients with psoriasis and atopic or allergic contact dermatitis and plays a key role in T-cell-mediated skin inflammation (Homey et al., 2002). Studies on the same datasets also showed that chemokines and their ligands were upregulated in AAA and could interact with other factors to play a role in AAA. For example, CCL4L1, CCL3L3, CXCL1, CXCL2, CXCL13, and CCR7 were all highly expressed in AAA (Gäbel et al., 2017; Gan et al., 2019). In one study, GNG2, CXCL1, and CCR7 were considered as central genes in the AAA network, and GNG2 interacted with CXCL1 and CCR7 to participate in the chemokine signaling road (Gan et al., 2019). PI3 is an important mediator in the occurrence and development of inflammation and synthesized and secreted by infiltrating neutrophils. Lipopolysaccharide, elastase, and TNF-α can also promote its production, and although the expression of PI3 is increased in the airway and mucosa where inflammatory stimulation persists (Sallenave et al., 1994; Pfundt et al., 1996, 2000; Reid et al., 1999), this does not mean that PI3 is a pro-inflammatory factor. On the contrary, PI3 seems to play a protective role in inflammation; for example, it is downregulated in the acute phase of acute respiratory distress syndrome, and in an experimental study, the plasma PI3 levels of control subjects were much higher than those of patients with respiratory distress syndrome. Therefore, a decrease in PI3 expression may lead to a decrease in the body’s tolerance for inflammation (Tejera et al., 2009). The mutation of *MAP2K1* is considered the most common cause of extracranial arteriovenous malformations. *MAP2K1* encodes MAP-extracellular signal-regulated kinase 1 (MEK1) and affects cell development through the RAS/MAPK signaling pathway. Extracranial arteriovenous malformations are characterized by vascular endothelial cell dysfunction and the promotion of arteriovenous malformations (Couto et al., 2017; Konczyk et al., 2020). These findings are mostly consistent with our results.

In our study, Gene Ontology and Kyoto Encyclopedia of Genes and Genomes analyses of differentially expressed genes showed that the upregulated genes were mainly involved in growth hormone response, peptide hormone response, growth factor activity, cytokine receptor activity, positive regulation of JAK-STAT cascade, and hormone-mediated signaling. Downregulated genes were mainly involved in T cell activation, negative regulation of external stimulus–response, response to TNF, cell chemotaxis, regulation of immune response molecular mediators, T cell activation, and leukocyte migration and proliferation. These biological processes and pathways are closely related to inflammation and immunity. Other studies on AAA have also confirmed that the interaction between cytokines and cytokine receptors, chemokine signaling pathway and T cell receptor signaling pathway play an important role in the occurrence and development of AAA (Biros et al., 2015). Therefore, we believe that the five immune response-related genes, *SSTR1, GPER1, CCR10, PI3*, and *MAP2K1*, may play important roles in the formation of AAA.

This study has the limitation of a small sample size for the measurement of selected gene expression; indeed, this may be the reason why we found no significant difference in PI3 expression. Hence, we plan to perform more in-depth studies on the selected genes to elucidate the mechanisms of action of these genes in the development of AAA.

## Conclusion

In this study, by analyzing an AAA microarray, we found that normal aorta and AAA samples could be clearly distinguished via the infiltration of immune cells and identified the key differentially expressed immune response-related genes, SSTR1, GPER1, CCR10, PI3, and MAP2K1.

Based on our results and the literature, we hypothesize that these five genes participate in the immune response and play important roles in the development of AAA. Thus, these genes may be key targets for the diagnosis and treatment of AAA.

## Data Availability Statement

Publicly available datasets were analyzed in this study. This data can be found here: GSE57691 (https://www.ncbi.nlm.nih.gov/geo/query/acc.cgi?acc=GSE57691) and GSE98278 (https://www.ncbi.nlm.nih.gov/geo/query/acc.cgi?acc=GSE98278).

## Ethics Statement

The studies involving human participants were reviewed and approved by the Medical Ethics Committee of the Second Affiliated Hospital of Nanchang University. The patients/participants provided their written informed consent to participate in this study.

## Author Contributions

HN: designing research direction and writing the manuscript. JQ: searching for references and writing the manuscript. SW: helping to revise manuscript. WZ: reviewing and revising the manuscript and guidance for manuscript preparation. All authors contributed to the article and approved the submitted version.

## Conflict of Interest

The authors declare that the research was conducted in the absence of any commercial or financial relationships that could be construed as a potential conflict of interest.

## Footnotes

1 http://www.ncbi.nlm.nih.gov/geo/

2 https://www.immport.org/home

3 https://string-db.org

4 http://www.cytoscape.org/

5 https://pga.mgh.harvard.edu/primerbank/

## Supplementary Material

Supplementary Figure 1 **(A)** Upregulation gene network map downloaded from STRING. **(B)** Downregulation gene network map downloaded from STRING.

Supplementary Figure 2 The expression of IL-6, IL-17 and TNF-α in the samples was analyzed using PCR (^∗^ represents *P* < 0.05).
