# Supplementary figures and images for: Combining Bioinformatics Techniques to Study the Key Immune-Related Genes in Abdominal Aortic Aneurysm
Source: Front Genet. 2020 Dec 10;11:579215. doi: 10.3389/fgene.2020.579215 (PMC7758434; doi:10.3389/fgene.2020.579215)

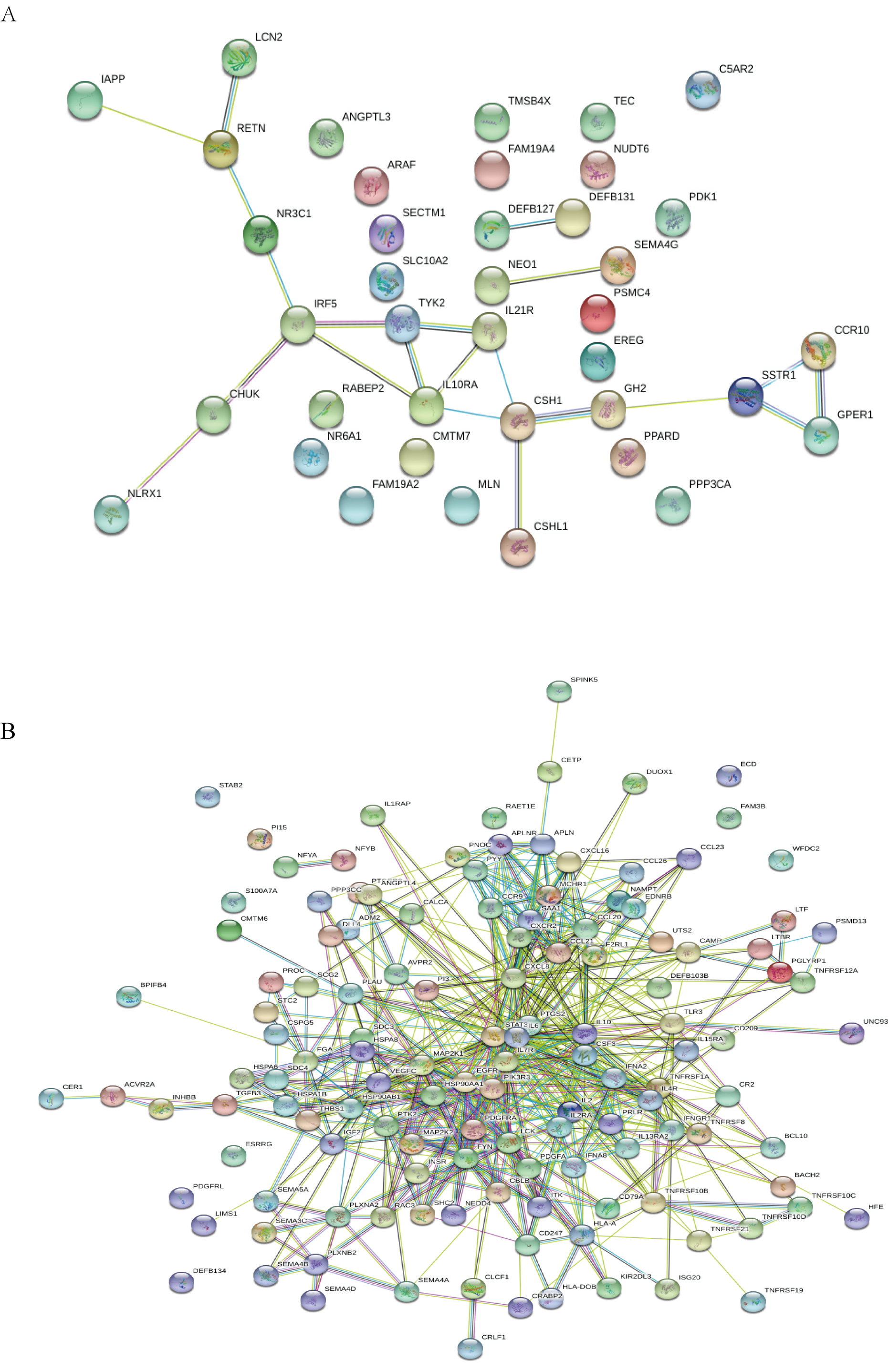

Supplement: Supplementary Figure 1 — (A) Upregulation gene network map downloaded from STRING. (B) Downregulation gene network map downloaded from STRING. [file Image_1.TIF]

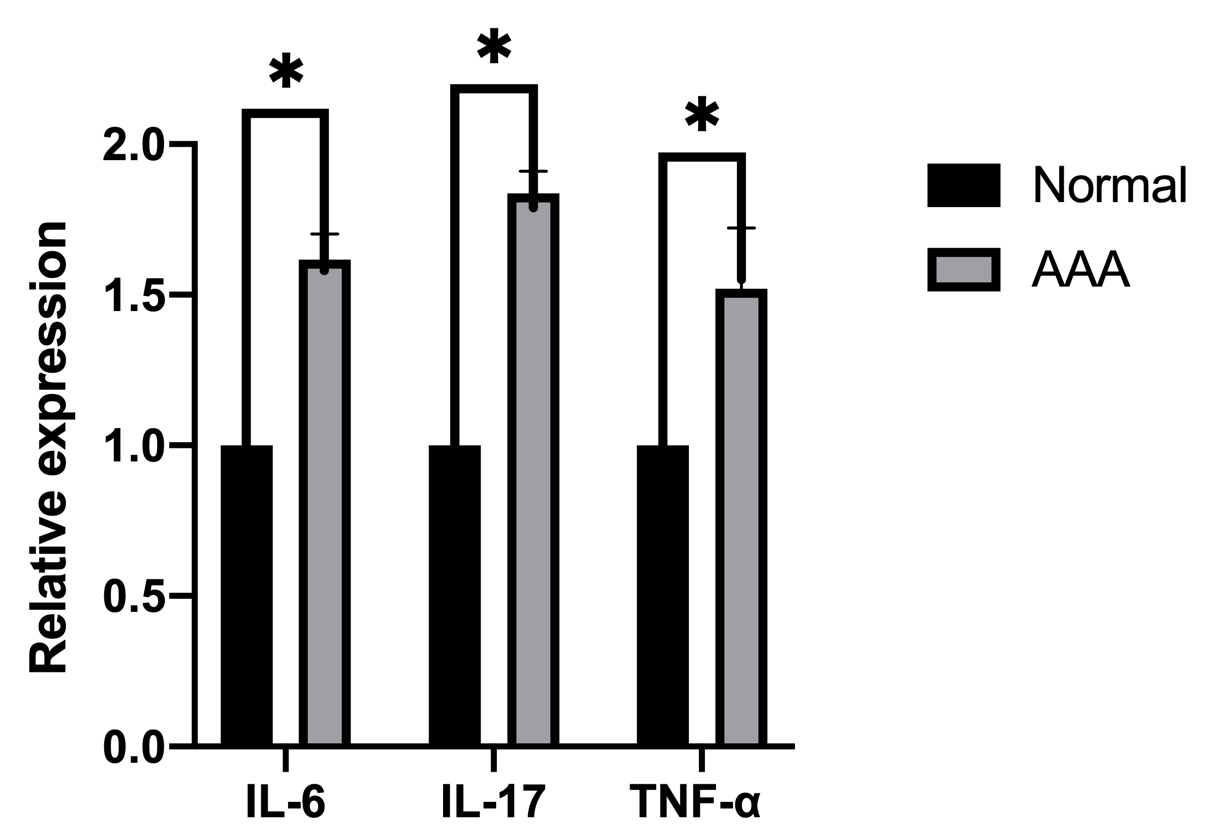

Supplement: Supplementary Figure 2 — The expression of IL-6, IL-17 and TNF-α in the samples was analyzed using PCR (∗ represents P < 0.05). [file Image_2.TIFF]
